# Comparative effectiveness of alternative spontaneous breathing trial techniques: a systematic review and network meta-analysis of randomized trials

**DOI:** 10.1186/s13054-024-04958-4

**Published:** 2024-06-08

**Authors:** Karen E. A. Burns, Behnam Sadeghirad, Maryam Ghadimi, Jeena Khan, Vorakamol Phoophiboon, Vatsal Trivedi, Carolina Gomez Builes, Benedetta Giammarioli, Kimberley Lewis, Dipayan Chaudhuri, Kairavi Desai, Jan O. Friedrich

**Affiliations:** 1 Departments of Critical Care Medicine and Medicine, Unity Health Toronto, St. Michael’s Hospital, 30 Bond Street, 4-045 Donnelly Wing, Toronto, ON M5B 1W8 Canada; 2 Interdepartmental Division of Critical Care Medicine, University of Toronto, Toronto, Canada; 3 Li Ka Shing Knowledge Institute, Unity Health Toronto, St. Michael’s Hospital, Toronto, Canada; 4 Department of Health Research Methods, Evidence, and Impact, McMaster University, Hamilton, Canada; 5 Departments of Anesthesia and Medicine, McMaster University, Hamilton, Canada; 6 Royal College of Surgeons of Ireland, University of Medicine and Health Sciences, Dublin, Ireland; 7 Division of Critical Care Medicine, Department of Medicine, Faculty of Medicine, Chulalongkorn University, Bangkok, Thailand; 8 Department of Anesthesiology & Pain Medicine, University of Toronto, Toronto, ON Canada; 9 Institute for Better Health, Trillium Health Partners, Mississauga, Canada; 10 Department of Critical Care, St. Joseph’s Healthcare, Hamilton, Canada; 11 Michael DeGroote School of Medicine, McMaster University, Hamilton, Canada

**Keywords:** Weaning, Spontaneous breathing trial, Extubation, Reintubation, Network meta-analysis

## Abstract

**Background:**

The spontaneous breathing trial (SBT) technique that best balance successful extubation with the risk for reintubation is unknown. We sought to determine the comparative efficacy and safety of alternative SBT techniques.

**Methods:**

We searched Medline, EMBASE, and the Cochrane Central Register of Controlled Trials from inception to February 2023 for randomized or quasi-randomized trials comparing SBT techniques in critically ill adults and children and reported initial SBT success, successful extubation, reintubation (primary outcomes) and mortality (ICU, hospital, most protracted; secondary outcome) rates. Two reviewers screened, reviewed full-texts, and abstracted data. We performed frequentist random-effects network meta-analysis.

**Results:**

We included 40 RCTs (6716 patients). Pressure Support (PS) versus T-piece SBTs was the most common comparison. Initial successful SBT rates were increased with PS [risk ratio (RR) 1.08, 95% confidence interval (CI) (1.05–1.11)], PS/automatic tube compensation (ATC) [1.12 (1.01 –1.25), high flow nasal cannulae (HFNC) [1.07 (1.00–1.13) (all moderate certainty), and ATC [RR 1.11, (1.03–1.20); low certainty] SBTs compared to T-piece SBTs. Similarly, initial successful SBT rates were increased with PS, ATC, and PS/ATC SBTs compared to continuous positive airway pressure (CPAP) SBTs. Successful extubation rates were increased with PS [RR 1.06, (1.03–1.09); high certainty], ATC [RR 1.13, (1.05–1.21); moderate certainty], and HFNC [RR 1.06, (1.02–1.11); high certainty] SBTs, compared to T-piece SBTs. There was little to no difference in reintubation rates with PS (vs. T-piece) SBTs [RR 1.05, (0.91–1.21); low certainty], but increased reintubation rates with PS [RR 2.84, (1.61–5.03); moderate certainty] and ATC [RR 2.95 (1.57–5.56); moderate certainty] SBTs compared to HFNC SBTs.

**Conclusions:**

SBTs conducted with pressure augmentation (PS, ATC, PS/ATC) versus without (T-piece, CPAP) increased initial successful SBT and successful extubation rates. Although SBTs conducted with PS or ATC versus HFNC increased reintubation rates, this was not the case for PS versus T-piece SBTs.

**Supplementary Information:**

The online version contains supplementary material available at 10.1186/s13054-024-04958-4.

## Background

For intubated critically ill adults and children, clinicians strive to reduce patients’ exposure to invasive mechanical ventilation to limit development of intubation and ventilator-related complications [1]. Identification of the earliest time that patients can resume spontaneous breathing is expected to reduce the time to successful extubation and thereby the duration of invasive ventilation [2, 3]. Simultaneously, clinicians must ensure that extubation does not increase the chance that critically ill patients will require reintubation. The risk of reintubation overall is approximately 10% but may be higher in selected populations including those who are at high risk of extubation failure [4].

Current clinical practice guidelines recommend systematically performing a spontaneous-breathing trial (SBT) before extubation [5]. A SBT is a focused assessment of patient’s capacity to breathe with either low levels or no ventilator support for a brief period of time [6]. Although conducted with an endotracheal tube in-situ, SBTs aim to assess readiness for extubation by simulating physiologic condition after extubation. SBTs can be performed using a variety of techniques that offer variable amount of inspiratory assistance and/or expiratory assistance. An international survey of stated practices in liberating critically ill patients from ventilators and a large observational study of actual liberation practices identified that pressure support (PS) with positive end-expiratory pressure (PEEP) and T-piece were the 2 most commonly used SBT techniques [7, 8]. Although a clinical meta-analysis suggested that significantly more patients were successfully extubated with a PS versus T-piece SBT, a concurrently conducted physiologic meta-analysis found that work of breathing during a PS trial was markedly lower than that needed during a T-piece SBT and after extubation [9, 10]. Concerns remain as to whether PS SBTs, while increasing rates of successful extubation, may increase the risk for reintubation due to underestimation of postextubation work of breathing [9, 11]. At present, considerable uncertainty exists regarding the best SBT for clinicians to use in clinical practice.

Most randomized controlled trials have compared the two most commonly used techniques, PS and T-piece. In a recent pairwise meta-analysis, including trials that compared 13 alternative SBT techniques, we identified that patients undergoing PS versus T-piece SBTs were 9% (95% CI, 6–12%) more likely to pass an SBT (after exclusion of an outlier trial with discordant effects on SBT and extubation outcomes) and 7% (95% CI, 4–10%) more likely to be successfully extubated without an increase in reintubation rate [12]. Since pair-wise meta-analysis only includes direct comparisons and reintubation was less commonly reported compared to SBT and extubation outcome, we sought to clarify the effects of alternative SBT techniques using direct and indirect evidence on important outcomes including SBT outcome (success vs. failure), successful extubation (success vs. failure), reintubation, and mortality.

## Methods

### Data sources and search strategy

An experienced health sciences librarian searched three databases utilizing database specific strategies without language restrictions (Medline, EMBASE, and the Cochrane Central Register of Controlled Trials) from inception through February 2023 to identify potentially eligible trials. We used the optimally sensitive search strategies for MEDLINE, EMBASE and the Cochrane Collaboration [13–15]. Additionally, 3 authors (VP, VT, JOF) hand-searched conference proceedings of 5 scientific meetings from 1990–April 2023: American Thoracic Society, American College of Chest Physicians (except 1999–2002, unavailable), International Symposium of Intensive Care and Emergency Medicine, European Society of Intensive Care Medicine, and Society of Critical Care Medicine, where feasible to April 2023. Ethics approval was not required. A registered protocol (PROSPERO CRD42023466265) guided conduct of the network meta-analysis.

### Study selection

Pairs of reviewers (VP, VT, CGB, BG, KL, DC, KD, JOF) independently screened citation titles, abstracts, and assessed full-text versions of potentially relevant trials. We included randomized or quasi-randomized trials that compared two or more SBT techniques in critically ill children and adults and reported at least one clinical important outcome including initial SBT or extubation outcome (success or failure), reintubation, time to first successful SBT, time to extubation or successful extubation, ventilator-associated pneumonia, intensive care unit (ICU) or hospital length of stay (LOS), mortality, post-extubation use of noninvasive ventilation (NIV) and high flow nasal cannula (HFNC), total duration of ventilation or adverse events as defined by the authors. We excluded trials that evaluated SBTs as part of a weaning strategy; neonatal patients, or tracheostomized patients (who do not undergo focused assessments using SBTs but rather tracheostomy mask trials); and trials evaluating automated SBTs (e.g., SmartCare,™ Intellivent®), NIV, and SBT versus no SBT. Two authors (KEAB, JOF) independently selected trials that met inclusion criteria and adjudicated disagreements.

The critical outcomes of interest for the network meta-analysis were SBT outcome (success/failure), extubation outcome (success/failure), and reintubation. Additional outcomes of interest included ICU mortality, hospital mortality, and the most protracted mortality reported by trial authors.

### Data extraction

Two investigators (KEAB, JOF) independently abstracted information regarding study characteristics, interventions, and data on outcomes of interest using a predesigned data extraction form and resolved disagreements through discussion [16].

### Risk of bias assessment

Two reviewers (KEAB, JOF) independently assessed the risk of bias (RoB) (including allocation concealment, randomization, blinded outcomes assessment, selective outcomes reporting, completeness of follow-up, stopping early for benefit). We judged each criterion for each trial as yes, no, unclear and assigned an overall RoB rating (high, unclear, low) [17]. As almost no trials had blinded outcome assessment, we focused on allocation concealment and incomplete outcome reporting in assessing each trial’s risk of bias. Reviewers resolved disagreements through discussion.

### Data synthesis and analysis

For all direct comparisons with at least two trials available for pooling, we performed random-effects meta-analysis for all outcomes and explored heterogeneity using the I^2^ statistic [18, 19] and visual inspection of forest plots. We categorized heterogeneity into intervals of 0%-40% (potentially negligible), 30%-60% (moderate), 50%-90% (significant), and 75% or more (considerable) [18, 19]. For pairwise meta-analyses, we calculated and reported risk ratio (RRs) for dichotomous outcomes with corresponding 95% confidence intervals (CIs). We performed Egger’s tests to assess for small-study effects when 10 or more trials were available for comparison [20].

To assess the feasibility of performing network meta-analysis, we ascertained that all SBT techniques were jointly randomizable, the network of evidence was connected for each outcome of interest, and the number of trials available for each network was more than the number of interventions [21, 22]. We used the ‘design-by-treatment’ model to assess the coherence assumption (consistency) for each network and the side-splitting method to evaluate local (loop-specific) incoherence [23–25]. We used a frequentist contrast-based random-effects model for network meta-analysis using the methodology of multivariate meta-analysis assuming a common heterogeneity parameter [26, 27]. For each outcome, we also estimated ranking probabilities using the surface under the cumulative ranking curve (SUCRA) and mean treatment rankings.

For the primary analysis, we evaluated the comparative efficacy and safety of alternative SBT techniques as nodes on important outcomes [SBT success, successful extubation, reintubation (primary outcomes)] and mortality (ICU, hospital, most protracted) with T-piece SBTs as the reference category.

In a planned sensitivity analysis we excluded one trial with internally inconsistent SBT and extubation outcome findings. We performed random-effects network meta-regression to investigate the impact of overall risk of bias (low versus unclear/high) on our findings.

### Assessing certainty of the evidence

We rated the certainty of evidence for each network estimate using the grading of recommendations, assessment, development, and evaluation (GRADE) framework, which classifies evidence as high, moderate, low, or very low certainty [28]. Two experienced reviewers (KEAB, JOF), familiar with GRADE assessments, rated the certainty of evidence for each direct comparison considering risk of bias, inconsistency, indirectness, and publication bias. We rated the certainty in none-zero (null) effect and when the point estimate was close to zero, we changed our target to trivial or no effect. We considered a minimally important difference of 3–4% to be important in network estimates [28–30].

Indirect effect estimates were calculated from available loops of evidence, which included first order loops (based on a single common comparator treatment—that is the difference between treatment A and B is based on comparisons of A and C as well as B and C) or higher order loops (more than one intervening treatment connecting the two interventions). We assessed the evidence for indirect estimates focusing on the dominant first order loop or, in the absence of a first order loop, a higher order loop [28] and rating certainty of indirect evidence as the lowest certainty of the contributing direct comparisons informing that dominant loop. We considered further rating down each indirect comparison for intransitivity if the distribution of effect modifiers differed in the contributing direct comparisons [28].

For the network estimate, we started with the certainty of evidence from the direct or indirect evidence that dominated the evidence and, subsequently, considered rating down our certainty in the network estimate for incoherence between the indirect and direct estimates, and for imprecision (wide credible intervals) around the treatment effect estimates. When serious incoherence was present, we used, as the best estimate, that with the higher certainty of the direct and indirect evidence [31].

## Results

### Search strategy and trial identification

**Fig. 1:**
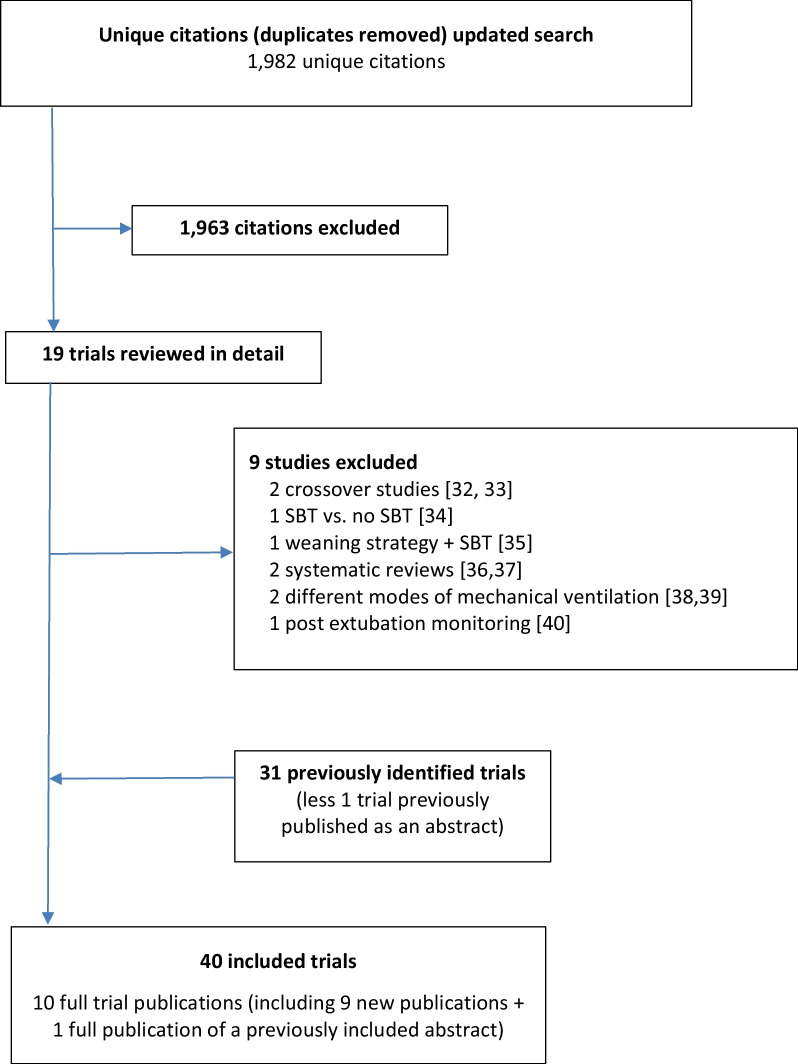
Identification of Trials included in the Network Meta-Analysis. SBT = spontaneous breathing trial

We identified 1,982 new unique citations (Fig. 1). Of these, 19 studies were assessed further for eligibility. We excluded 9 studies [32–40]. In addition to the previously identified 31 trials [41–71], we identified 10 additional trials [72–80] (one of which was a full publication [73] of a previously published abstract) for inclusion; the 9 new trials reported on 3,130 patients. In total, we included 40 trials reporting on 6,716 patients.

### Study characteristics, risk of *bias*, and certainty of evidence

The characteristics of the included trials are presented in Table 1, Additional file 1: Table S1. Among the 40 included trials, 6 trials [46, 47, 52, 53, 69, 77] compared 3 SBT techniques (though for one of these 3-arm trials [47] we combined the two 5 cm H_2_O and 10 cm H_2_O continuous positive airway pressure (CPAP) arms for a single comparison to the T-piece arm) and 1 trial [71] compared 4 SBT techniques. Two trials [61, 72] appeared to be published, at least in part, in duplicate [81, 82]. Risk of bias assessments for each included trial are depicted in Additional file 1: Table S2.

**Table 1:** Characteristics of included trials

Trial characteristics	Number of trials (%)	Number of patients (%)
Continent of study		
Europe (including United Kingdom)	9 (22.5%)	2855 (43%)
Asia	6 (15%)	1241 (18%)
Middle East (including Turkey)	11 (27.5%)	932 (14%)
North America	6 (15%)	359 (5%)
South America	6 (15%)	588 (9%)
Europe (Spain) & South America	2 ( 5%)	741 (11%)
Total	40 (100%)	6,716 (100%)
Year of publication		
Before 1990	4 (10%)	104 (2%)
1990–1994	2 ( 5%)	168 (3%)
1995–1999	3 ( 7.5%)	594 (9%)
2000–2004	5 (12.5%)	702 (10%)
2005–2009	7 (17.5%)	870 (13%)
2010–2014	10 (25%)	633 (9%)
2015–2019	6 (15%)	2462 (37%)
2020–2023	3 ( 7.5%)	1183 (18%)
Total	40 (100%)	6716 (100%)
Patient population		
Adults	37 (92.5%)	6372 (95%)
Children	3 (7.5%)	344 (5%)
Total	40 (100%)	6716 (100%)
Type of ICU		
Medical-Surgical	10 (25%)	1628 (24%)
General/Not Specified	11 (27.5%)	3088 (46%)
Medical	5 (12.5%)	451 (7%)
Respiratory	1 (2.5%)	166 (3%)
Coronary	1 (2.5%)	120 (2%)
Surgical	3 (7.5%)	702 (10%)
Cardiac-Surgical	6 (15%)	217 (3%)
Pediatric Intensive Care Unit	3 (7.5%)	344 (5%)
Total	40 (100%)	6716 (100%)
Alternative SBT Technique Comparisons		
T-piece versus PS	18 (33%)	4862 (64%)
T-piece versus CPAP	10 (18%)	528 (7%)
T-piece versus ATC	4 (7%)	267 (4%)
T-piece versus HFNC	3 (5%)	386 (5%)
T-piece versus PAV+	1 (2%)	118 (2%)
T-piece versus IMV	1 (2%)	40 (0.5%)
PS versus CPAP	3 (5%)	130 (2%)
PS versus ATC	5 (9%)	502 (7%)
PS versus PAV+	1 (2%)	96 (1%)
PS versus HFNC	1 (2%)	178 (2%)
PS versus PS/ATC	1 (2%)	100 (1%)
CPAP versus ATC/CPAP	2 (4%)	129 (2%)
CPAP versus ATC	2 (4%)	178 (2%)
CPAP versus IMV	2 (4%)	59 (0.8%)
IMV versus SVT	1 (2%)	28 (0.4%)
Total	55 (100%)*	7601 (100%)

SBT = spontaneous breathing trial, PS = pressure support, T-piece = T tube, ATC = automatic tube compensation, PS/ATC = pressure support ventilation/automatic tube compensation, CPAP = continuous positive airway pressure, HFNC = high flow nasal cannulae, IMV = invasive mechanical ventilation, PAV +  = proportional assistance ventilation plus, SVT = spontaneous ventilation

*Includes 5 trials with 3 groups (3 comparisons) and 1 trial with 4 groups (6 comparisons) for a total of 15 additional comparisons. For a 6th trial with 3 groups (CPAP 5 cm H_2_O vs CPAP 10 cm H_2_O vs T-piece), we combined the two CPAP groups for a single comparison versus T-piece

### Outcomes

#### Initial SBT success

**Fig. 2:**
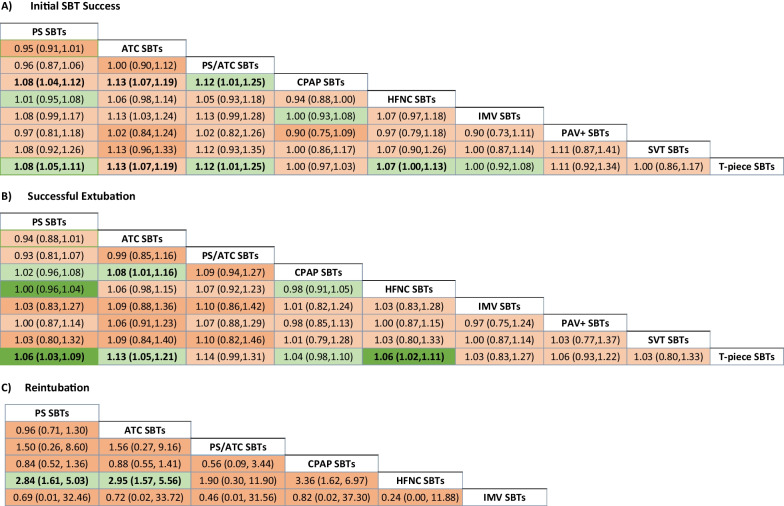
Network Estimates and Certainty Ratings for Alternative SBT Techniques on Primary Outcomes. SBT = spontaneous breathing trial, PS = pressure support, ATC = automatic tube compensation, PS/ATC = pressure support ventilation/automatic tube compensation, CPAP = continuous positive airway pressure, HFNC = high flow nasal cannulae, IMV = invasive mechanical ventilation, PAV+  = proportional assistance ventilation plus, SVT = spontaneous ventilation, T-piece = T tube. Spontaneous breathing trial network meta-analysis results with corresponding GRADE (grading of recommendations, assessment, development, and evaluation) certainty of evidence (dark green , high certainty; light green, moderate certainty; light orange, low certainty; and dark orange, very low certainty) for **A** initial SBT success, **B** successful extubation and **C** reintubation rates excluding a single outlier trial (73). Values correspond to difference between columns and rows in the rate of **A** initial SBT success [excluding a single outlier trial (73)], **B** successful extubation and **C** reintubation. Values in bold indicate a statistically significant treatment effect

**Fig. 3:**
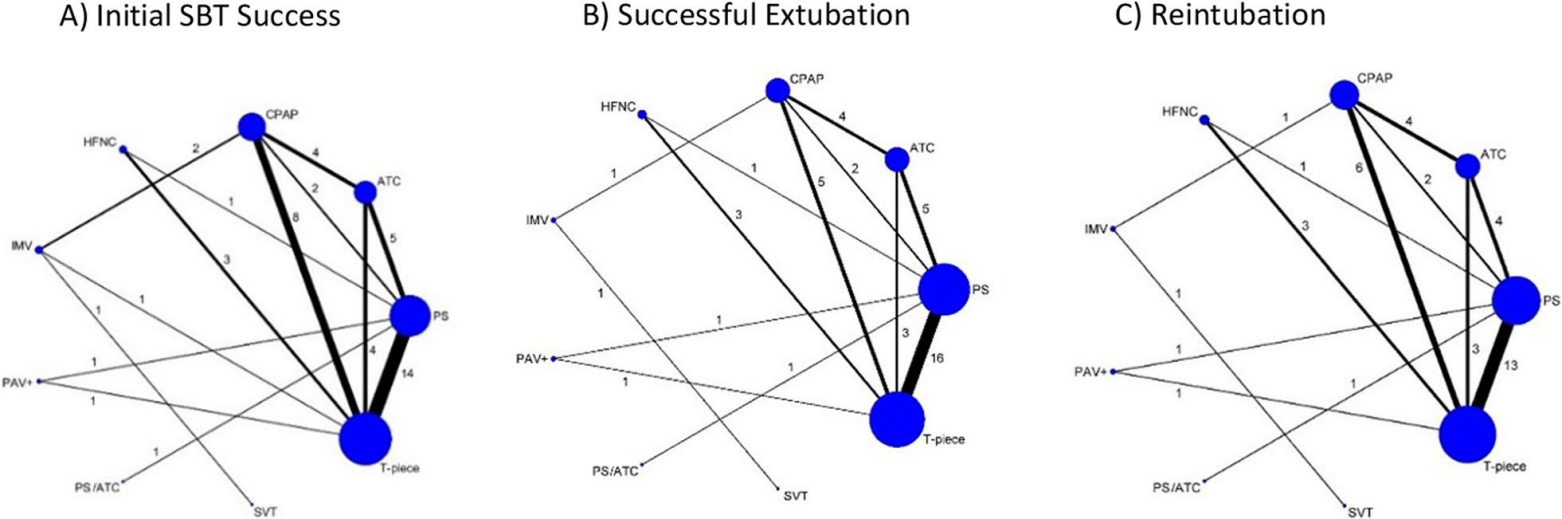
Network Plots. **A**–**C** Network plots **A** successful spontaneous breathing trial (SBT) **B** successful extubation **C** Reintubation. The size of the node corresponds to the number of patients randomized to that intervention. The thickness of the line and the associated numbers correspond to the number of studies comparing the two linked interventions. SBT = spontaneous breathing trial; CPAP = continuous positive airway pressure; ATC = automatic tube compensation; PSV = pressure support ventilation; HFNC = high flow nasal cannulae; IMV = intermittent mandatory ventilation; PAV+  = proportional assist ventilation plus; PSV/ATC = pressure support ventilation/automatic tube compensation; SVT = spontaneous ventilation

We present the comparative efficacy of the alternative SBT techniques on initial SBT success from 35 trials (including 48 comparisons), excluding a single outlier trial [73] with their associated GRADE certainty ratings in Fig. 2A. We considered this to be the primary analysis as heterogeneity was reduced from 73 to 0% following exclusion of this trial. The network plot for initial SBT success is shown in Fig. 3A. Surface under the cumulative ranking curve rankings are displayed in Additional file 1: Table S3 and the direct and indirect estimates with tests of incoherence for initial successful SBT are shown in Additional file 1: Table S4. Compared to T-piece SBTs, PS SBTs [RR 1.03, (95% CI 0.98–1.08); low certainty] may result in little or no difference in initial SBT success rates. However, when a single trial with internally inconsistent results between initial SBT and extubation outcomes was removed PS (vs. T-piece) SBTs likely increase initial SBT success rates [RR 1.08, 95% CI (1.05–1.11); moderate certainty].

Compared to T-piece SBTs, PS/automatic tube compensation (ATC) [1.12 (95% CI 1.01–1.25); moderate certainty] and HFNC [1.07 (95% CI 1.00–1.13); moderate certainty**]** SBTs increased the proportion of patients who passed an initial SBT. Similarly, compared to T-piece SBTs, ATC SBTs may increase initial SBT success rates [RR 1.13, (95% CI 1.07–1.19); low certainty]. Compared to CPAP SBTs, PS SBTs [RR 1.08, (95% CI 1.04–1.12); low certainty], and ATC SBTs [RR 1.13, (95% CI 1.07–1.19); low certainty] may increase initial SBT success rates and PS/ATC SBTs [RR 1.12, (95% CI 1.01–1.25); moderate certainty] likely increased initial SBT success rates.

#### Successful extubation

We present the comparative efficacy of the alternative SBT techniques on successful extubation from 31 trials (including 44 comparisons) with their associated GRADE certainty ratings in Fig. 2B. We depict the network plot for initial SBT success in Fig. 3B. Surface under the cumulative ranking curve rankings are displayed in Additional file 1: Table S5 and the direct and indirect estimates with tests of incoherence for successful extubation are shown in Additional file 1: Table S6.

In network estimates compared to T-piece SBTs, PS [RR 1.06, (95% CI 1.03–1.09; high certainty) and HFNC SBTs [RR 1.06, (95% CI 1.02–1.11); high certainty] increased the proportion of patients who were successfully extubated. Both ATC (vs. T-piece) SBTs [RR 1.13, (95% CI 1.05–1.21); moderate certainty] and ATC (vs. CPAP) SBTs [RR 1.08, (95% CI 1.01–1.16); moderate certainty] likely increased the proportion of successfully extubated patients.

#### Reintubation

We present the comparative efficacy of the alternative SBT techniques on reintubation from 30 trials (including 41 comparisons) with their associated GRADE certainty ratings in Fig. 2C. We depict the network plot for initial SBT success in Fig. 3C. Surface under the cumulative ranking curve rankings are displayed in Additional file 1: Table S7 and the direct and indirect estimates with tests of incoherence for reintubation are shown in Additional file 1: Table S8.

Both PS (vs. HFNC) SBTs [RR 2.84, (95% CI, 1.61–5.03); moderate certainty] and ATC (vs. HFNC) SBTs [RR 2.95 (95% CI, 1.57–5.56); moderate certainty) likely resulted in a large increase in the proportion of patients who were reintubated. PS (vs. T-piece) SBTs may result in little or no difference in reintubation rate [RR 1.05, (95% CI 0.91–1.21); low certainty].

Using a minimally contextualized framework [83], we summarize the comparative effectiveness and safety of the alternative SBT technique on primary outcomes in Table 2.

**Table 2:** Ranking tables for primary outcomes

Ranking classification	Initial successful SBT	Successful extubation	Reintubation
Among the best (moderate to high certainty)	PS PS/ATC HFNC	PS HFNC ATC	HFNC
Maybe among the best (low or very low certainty)	ATC	*PS/ATC	
No different than T-piece (moderate to high certainty)	IMV	CPAP	
Maybe no different than T-piece (low to very low certainty)	CPAP SVT PAV+	IMV SVT PAV+	PS PS/ATC ATC PAV+ IMV SVT CPAP

Ranking of SBT techniques using minimally contextualized framework [83] with T-piece SBTs serving as the reference framework

SBT = spontaneous breathing trial; PS = pressure support; PS/ATC = pressure support/automatic tube compensation; HFNC = high flow nasal cannulae; ATC = automatic tube compensation; IMV = intermittent mandatory ventilation; CPAP = continuous positive airway pressure; SVT = spontaneous ventilation; PAV+  = proportional assist ventilation plus

*PS/ATC did not achieve statistical significance [RR 1.14 (95% CI, 0.99, 1.31)] and therefore could arguably be placed in the ‘maybe not different than T-piece’ (low to very low certainty) classification

### Secondary outcomes

Network meta-analysis of alternative SBT techniques on the incidence of ICU (Additional file 1: Table S9, Additional file 1: Figure S1), hospital (Additional file 1: Table S10, Additional file 1: Figure S2), and most protracted mortality (Additional file 1: Table S11, Additional file 1: Figure S3) were not significant.

### Network meta-regression

For the comparison of ATC versus PS SBTs on initial SBT outcome [excluding an outlier trial (73)], the effect estimate for trials at low (vs. unclear/high) risk of bias was RR 1.10 (95% CI 1.03–1.18) (test of interaction *p*-value = 0.018) (Additional file 1: Table S12). Similarly, for the comparison of ATC (vs PS) SBTs on successful extubation, the effect estimate for trials at low (vs. unclear/high) risk of bias was RR 1.19 (95% CI 1.06–1.34) (test of interaction *p*-value = 0.026). (Additional file 1: Table S13). There were no significant tests of interaction between SBT techniques and risk of bias for reintubation (Additional file 1: Table S14).

## Discussion

This network meta-analysis included a large number of trials and well-connected network plots with many direct and indirect comparisons. The largest number of trials directly compared PS to T-piece SBTs. Using direct and indirect evidence, we identified that compared to T-piece SBTs, initial successful SBT rates were increased with PS (when an outlier trial was excluded), PS/ATC, and HFNC SBTs (all moderate certainty) and with ATC SBTs (low certainty). Compared to CPAP SBTs, 3 SBT techniques [PS (outlier excluded; low certainty), ATC (low certainty), and PS/ATC SBTs (moderate certainty); also increased initial successful SBT rates. Compared to T-piece SBTs, successful extubation rates were increased with PS and HFNC (both high certainty] and ATC (moderate certainty) SBTs. Successful extubation rates were also increased with ATC (vs. CPAP) SBTs (moderate certainty). There may be little to no difference in reintubation with PS (vs. T-piece) SBTs (low certainty), but reintubation rates were likely increased with ATC SBTs (low certainty) and PS SBTs (moderate certainty)] compared to HFNC SBTs. Taken together, network meta-analysis favored use of SBT techniques with pressure augmentation (PS, ATC, PS/ATC) versus without (T-piece, CPAP) for successful initial SBT and extubation rates (indirect evidence only, moderate certainty). There may be a trade-off between pressure augmentation and reintubation risk in PS and ATC (vs. HFNC) SBTs (direct evidence, moderate certainty), although data suggest there may be little to no difference in reintubation rate with PS (vs. T-piece) SBTs; (direct evidence; low certainty).

In the absence of a large equivalency trial comparing alternative SBT techniques, the best SBT technique for clinicians to utilize in practice remains unclear. Consequently, considerable international practice variation exists in the conduct of SBTs [8]. For patients who are invasively ventilated for > 24 h, the American Thoracic Society/American College of Chest Physicians guideline [5] provided a conditional recommendation (moderate certainty) to conduct SBTs with inspiratory pressure augmentation of 5–8 cm H_2_O versus without pressure augmentation (vs. T-piece or CPAP) [5]. Our findings align with these guideline recommendations. Network meta-analysis identified that both PS, ATC, and PS/ATC SBTs increased successful initial SBT rates and PS (vs. T-piece) and ATC SBTs (vs. T-piece and CPAP) increased successfully extubation rates. Additionally, we identified that HFNC (vs. T-piece) SBTs increased both initial successful SBT and extubation rates. Of these comparisons, the largest amount of direct evidence emanated from trials that compared PS (vs. T-piece). Compared to T-piece SBTs, PS SBTs likely increased the proportion of initial SBT successes (moderate certainty) and increased the rate of successful extubation (high certainty), and may result in little difference in reintubation rate (low certainty). Data supporting comparisons between ATC (vs. T-piece) and ATC (vs. CPAP) SBTs on initial successful SBT and extubation were enhanced by indirect evidence from the large number of trials that compared PS and T-piece SBTs. Similarly, direct evidence comparing HFNC (vs. T-piece) SBTs emanated from only 3 trials and were similarly enhanced by indirect evidence from PS comparisons.

One of the novel findings of this network meta-analysis was the likely higher reintubation rates associated with both PS and ATC SBTs (vs. HFNC; both moderate certainty) conducted with augmented inspiratory support and the lower reintubation rate associated with HFNC versus T-piece (moderate certainty). There are several reasons why the effect estimates of alternative SBT comparisons on reintubation rates were different. First, reintubation was less frequently reported (vs. initial SBT success and successful extubation) as a trial outcome and certainty of network estimates for reintubation (Fig. 2C) were lower than for successful initial SBT (Fig. 2A) and extubation (Fig. 2B). Second, HFNC data reflecting reintubation rate emanated from only 3 trials (n = 482) [66, 67, 72] that contributed data to 4 comparisons. Of these, a single three arm trial [66] included most patients (n = 268) and contributed to 2 HFNC pairwise comparisons. This trial [66] also had unclear risk of bias with regard to random sequence generation, allocation concealment, completeness of outcomes reporting, and early stopping. Third, the reintubation network figure (Fig. 3C) shows that only one trial directly compared PS versus HFNC SBTs and no trial directly compared ATC versus HFNC SBTs. Consequently, the evidence supporting these findings is largely indirect. By contrast, 13 trials directly compared PS versus T-piece SBTs and reported reintubation rates. On balance, although the inferences that can be made from PS and ATC (vs. HFNC) SBTs were limited, a large number of trials, though with only low certainty evidence, supported that there may be little to no difference in reintubation rates with PS (vs. T-piece) SBTs. Taken together, the network meta-analysis supports use of PS (vs. T-piece) SBTs with a significantly higher successful extubation rate and similar reintubation rate. To address the potential trade-off, a large well-designed trial powered to assess reintubation rates, would be required to clarify the effect of SBTs with (vs. without) pressure augmentation.

A single SBT technique is unlikely to be optimal for all intubated patients. Prior research has similarly identified that compared to T-piece, PS SBTs may offset clinician reluctance to extubate, thereby enabling timely and more successful extubation decision-making [9, 12, 84, 85]. Although combining direct and indirect evidence from randomized trials permitted comparisons between multiple SBT techniques, several points should be considered in interpreting our findings. First, many participants in the included trials likely had a high pretest probability of passing an SBT and being successfully extubated after an initial SBT [86]. Second, T-piece SBTs may be appropriate, even ideal, for specific patients including those with left ventricular dysfunction, neuromuscular weakness, or marginal reserve). T-piece SBTs may also be preferred when clinicians are uncertain about SBT or extubation outcomes and therefore prioritize a technique with a low false positive rate to limit the likelihood of extubation failure [4, 9]. However, use of T-piece SBTs for all critically patients, including patients with a high pretest probability of success, may lead to a high false negative rate and result in patients remaining on invasive ventilation longer than needed. To this end, most trials were conducted in medical, surgical, or mixed populations with limited data emanating from specific populations. Third, the included trials differed in how often the assigned SBT techniques were used with few trials applying interventions until a clinical outcome (successful extubation, death, transfer or discharge) was achieved. Fourth, successful extubation incorporates both the ability to pass a SBT and remain extubated and was variably defined in the included trials. In modern day practice, successful extubation may be influenced by post-extubation use of bilevel NIV, CPAP, or HFNC [87].

Our review has strengths. We conducted a comprehensive literature search, performed duplicate eligibility appraisal, risk of bias assessment, and data abstraction. We conducted meta-regression to account for potential effect modifiers (risk of bias) and used GRADE to rate certainty of evidence. Inclusion of a large number of trials, enabled creation of well-linked and connected network plots with many direct and indirect comparisons. Our review also has several important limitations. First, there was not enough direct evidence for PS and ATC (vs. HFNC) and, to a lesser extent, PS (vs. T-piece) comparisons on reintubation rate to make strong inferences. Second, we identified only 3 trials involving critically ill children where considerable uncertainty still exists regarding the role for SBTs in liberation from invasive ventilation. Third, there may be unknown and unmeasured confounders that could have impacted the intransitivity assumption in assessing certainty of the evidence. Fourth, publication bias may have impacted our findings as few comparisons included more than ten trials. Finally, we did not involve patients or family members in the design or conduct of this study. Notwithstanding, we highlighted liberation and general outcomes that are important to visitors to ICUs in our primary and secondary outcomes [88].

## Conclusions

SBTs conducted with pressure augmentation (PS, ATC, PS/ATC) versus without (T-piece, CPAP) increased initial successful SBT and successful extubation rates. Although SBTs conducted with PS or ATC compared to HFNC increased reintubation rates, this was not the case for PS versus T-piece SBTs.

### Supplementary Information

Additional file 1 (DOCX 343 kb)

## Data Availability

The search strategy, template data collection forms, data extracted from included trials, and data used for analyses are available upon written request to Dr. Burns.

## Abbreviations

SBT: Spontaneous breathing trial
PS: Pressure support
PEEP: Positive end-expiratory pressure
ICU: Intensive care unit
LOS: Length of stay
NIV: Noninvasive ventilation
HFNC: High flow nasal cannulae (also known as high flow oxygen)
RoB: Risk of bias
RR: Relative risk
CI: Confidence interval
SUCRA: Surface under the cumulative ranking curve
GRADE: Grading of recommendations, assessment, development, and evaluation framework
CPAP: Continuous positive airway pressure
ATC: Automatic tube compensation

## Declarations

### Guarantor statement

KEAB, JOF and BS had full access to all the data in the study and take responsibility for the integrity of the data and the accuracy of the data analysis. The corresponding author (KB) attests that all listed authors met authorship criteria and that no others meeting the criteria have been omitted.
