# Supplementary material for: Comparative effectiveness of alternative spontaneous breathing trial techniques: a systematic review and network meta-analysis of randomized trials
Source: Crit Care. 2024 Jun 8;28:194. doi: 10.1186/s13054-024-04958-4 (PMC11162018; doi:10.1186/s13054-024-04958-4)

**Table S1: Characteristics of Included Trials**

| **Author Year**  **[n]** | **Population** | **Duration of Ventilation at Inclusion** | **Interventions** | **Country** | **Publication**  **Type** |
| --- | --- | --- | --- | --- | --- |
| **Feeley 1975 ^(41)^**  **[25]** | Adult | Not reported | T-piece/PEEP 5 cm H_2_O vs. T-piece | USA | Full |
| **Hastings 1980 ^(42)^**  **[18]** | Adult | Perioperative | IMV/CPAP 5 cm H_2_O vs.  T-piece/CPAP 5 cm H_2_O | USA | Full |
| **Prakash 1982 ^(43)^**  **[28]** | Adult | Perioperative | IMV vs. SVT (on ventilator) | Netherlands | Full |
| **Koller 1983 ^(44)^**  **[45]** | Adult | Perioperative | CPAP 10 cm H_2_O vs. T-piece/ZEEP | Austria | Full |
| **Jones 1991 ^(45)^**  **[106]** | Adult | Not reported | CPAP 5 cm H_2_O vs. T-piece/ZEEP | USA | Full |
| **Abalos 1992 ^(46)^**  **[62]** | Adult | Perioperative | SIMV vs. CPAP 4 cm H_2_O vs. T-piece | USA | Full |
| **Bailey 1995 ^(47)^**  **[82]** | Adult | Perioperative | T-piece/CPAP 0 cm H_2_O vs.  CPAP 5 cm H_2_O vs. CPAP 10 cm H_2_O | England | Full |
| **Schinco 1995 ^(48)^**  **[30]** | Adult | Perioperative | PS 5 cm H_2_O/CPAP 5 cm H_2_O vs. CPAP 5 cm H_2_O | USA | Abstract |
| **Esteban 1997 ^(49)^**  **[484]** | Adult | >48 h | T-piece vs. PS 7 cm H_2_O | Spain & South America | Full |
| **Holanda 2000 ^(50)^**  **[35]** | Adult | >48 h | T-piece vs. PS 7 cm H_2_O | Brazil | Abstract |
| **Farias 2001 ^(51)^**  **[257]** | Pediatric | >48 h | T-piece vs.  PS 10 cm H_2_O ±/ PEEP 5 cm H_2_O | Argentina | Full |
| **Haberthur 2002 ^(52)^**  **[90]** | Adult | >24 h | PS 5 cm H_2_O/PEEP 5 cm H_2_O vs. ATC/PEEP 5 cm H_2_O vs. T-piece | Switzerland | Full |
| **Koksal 2004 ^(53)^**  **[60]** | Adult | >48 h | PS < 10 cm H_2_O/PEEP < 5 cm H_2_O vs. CPAP < 5 cm H_2_O vs. T-piece | Turkey | Full |
| **Matic 2004 ^(54)^**  **[260]** | Adult | >48 h | T-piece vs. PS 8 cm H_2_O | Croatia | Full |
| **Cohen 2006 ^(55)^**  **[99]** | Adult | >24 h | ATC^†^/CPAP 5 cm H_2_O vs.  CPAP 5 cm H_2_O | Israel | Full |
| **Liang 2006 ^(56)^**  **[97]** | Adult | >4 days | ATC vs. T-piece | Taiwan | Abstract |
| **Colombo 2007 ^(57)^**  **[120]** | Adult | >48 h | T-piece vs.  PS 7 cm H_2_O/PEEP 5 cm H_2_O | Brazil | Full |
| **Matic 2007 ^(58)^**  **[136]** | Adult | >24 h | T-piece vs. PS (not specified) | Croatia | Full |
| **Fayed 2008 ^(59)^**  **[30]** | Adult | >24 h | ATC^†^/CPAP 5 cm H_2_O vs.  CPAP 5 cm H_2_O | Egypt | Abstract |
| **Cohen 2009 ^(60)^**  **[180]** | Adult | >24 h | ATC^†^/CPAP 5 cm H_2_O vs.  PS 7 cm H_2_O /CPAP 5 cm H_2_O | Israel | Full |
| **Zhang 2009 ^(61)^**  **[208]** | Adult | Not reported | T-piece vs.  PS 5 cm H_2_O/PEEP 5 cm H_2_O | China | Full |
| **Figueroa-Casas 2010 ^(62)^ [122]** | Adult | >24 h | ATC^†^/PEEP 5 cm H_2_O vs.  CPAP 5 cm H_2_O | USA | Full |
| **Molina-Saldarriaga 2010 ^(63)^ [50]** | Adult | >48 h | CPAP^‡^/PEEP permitted vs.  T-piece/ PEEP permitted | Colombia | Full |
| **Cekman 2011 ^(64)^**  **[40]** | Adult | >48 h | CPAP < 5 cm H_2_O vs. T-piece | Turkey | Full |
| **Vats 2012 ^(65)^**  **[40]** | Adult | Not reported | T-piece vs. PS 7 cm H_2_O | India | Full |
| **El-beleidy 2013 ^(66)^**  **[36]** | Pediatric | >24 h | ATC^†^/CPAP 5 cm H_2_O vs.  PS 6-10 cm H_2_O /CPAP 5 cm H_2_O | Egypt | Full |
| **Lourenco 2013 ^(67)^**  **[30]** | Adult | Perioperative | T-piece vs. PS (not specified) | Brazil | Full |
| **Sherif 2013 ^(68)^**  **[100]** | Adult | Not reported | PS (not specified) vs. PS/ATC | Egypt | Abstract |
| **Selek 2014 ^(72)^**  **[50]** | Adult | >24 h | T-piece under spontaneous breathing vs. ATC/CPAP ≤ 5 cm H_2_O | Turkey | Full |
| **Zanfaly 2014 ^(73)^**  **[120]** | Adult | >48 h | ATC/PEEP 5 cm H_2_O vs. PS 7 cm H_2_O vs. CPAP 5 cm H_2_O vs. T-piece | Egypt | Full |
| **Bilan 2015 ^(69)^**  **[51]** | Pediatric | Not reported | CPAP vs. T-piece | Iran | Full |
| **Chittawatanarat 2018 ^(70)^**  **[520]** | Adult | >12 h | T-piece vs.  PS 7 cm H_2_O/PEEP < 5 cm H_2_O | Thailand | Full |
| **Teixeira 2015 ^(71)^**  **[160]** | Adult | >24 h | PS 7 cm H_2_O vs. PAV+ vs. T-piece | Brazil | Full |
| **El-Shahat 2015 ^(74)^**  **[166]** | Adult | >24 h | PS/ PEEP 0­-5 cm H_2_O vs.  ATC/ PEEP 0­-5 cm H_2_O | Egypt | Full |
| **Santos Pellegrini 2018 ^(75)^**  **[190]** | Adult | >48 h | T-piece vs. PS 10 cm H_2_O | Brazil | Full |
| **Subirà 2019 ^(76)^**  **[1153]** | Adult | >24 h | T-piece (x2 h) vs. PS 8 cm H_2_O (30 min) | Spain | Full |
| **Liu 2019 ^(77)^**  **[268]** | Adult | >48 h | T-piece vs. PS 7 cm H_2_O vs. HFNC | China | Full |
| **Fossat 2021 ^(78)^**  **[106]** | Adult | >24 h | T-piece vs. HFNC | France | Full |
| **Thille 2022 ^(79)^**  **[969]** | Adult | >24 h | PS vs. T-piece | France | Full |
| **Lee 2022 ^(80)^**  **[108]** | Adult | >24 h | T-piece vs. HFNC (at end of ETT) | Korea | Full |

*Legend*

PEEP = positive end-expiratory pressure; IMV = intermittent mandatory ventilation; CPAP = continuous positive airway pressure; SVT = spontaneous ventilation trial, ZEEP = zero end-expiratory pressure; SIMV = synchronized intermittent mandatory ventilation; PS = pressure support; ATC = automatic tube compensation; PAV+ = proportional assist ventilation with load adjustable gain factors; USA = United States of America; HFNC = High flow nasal cannulae; ETT = endotracheal tube

^†^ATC with 100% compensation

^‡^ CPAP set to 85% of intrinsic PEEP

**Table S2: Risk of Bias of the Included Trials**


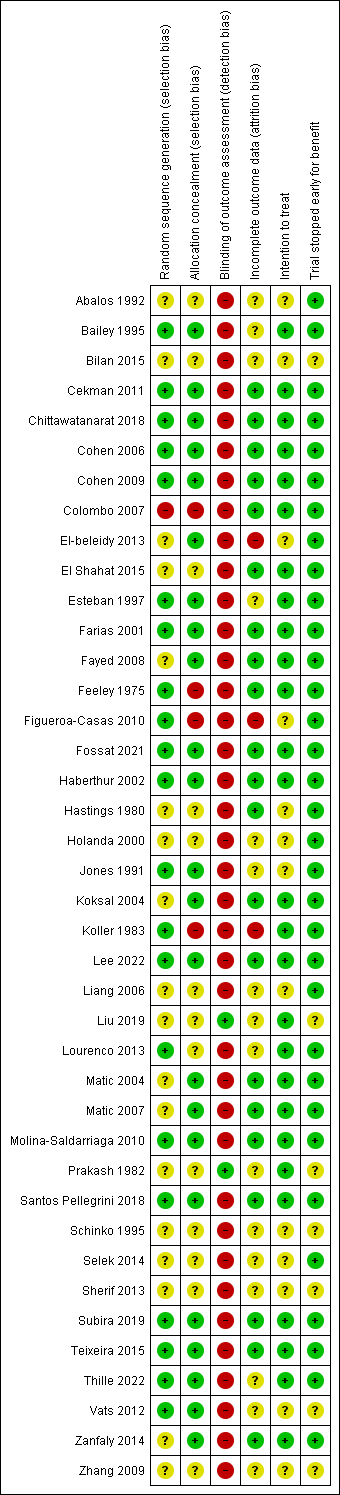


Figure Legend:

Figure depicts the risk of bias of the included trials.

**Table S3: SUCRA and Ranking Probabilities for Alternative SBTs on Initial SBT Success**

| Treatment | SUCRA | Probability of being the best (%) | Mean Rank |
| --- | --- | --- | --- |
| ATC | 86.1 | 28.8 | 2.1 |
| PS/ATC | 80.6 | 31.4 | 2.5 |
| PAV+ | 68.6 | 34.1 | 3.5 |
| PS | 62.8 | 0.2 | 4.0 |
| HFNC | 58.2 | 2.0 | 4.3 |
| SVT | 29.5 | 3.5 | 6.6 |
| CPAP | 21.5 | 0.0 | 7.3 |
| T-piece | 21.4 | 0.0 | 7.3 |
| IMV | 21.3 | 0.0 | 7.3 |

Legend

PS = pressure support; ATC = automatic tube compensation; PAV+ = proportional assist ventilation with load adjustable gain factors; HFNC = High flow nasal cannula; SVT = spontaneous ventilation trial; CPAP = continuous positive airway pressure; IMV = intermittent mandatory ventilation.

**Table S4 Direct and Indirect Estimates of Effect and Tests for Incoherence for Initial SBT Success**

| **Comparison** | **Direct ES** | **Indirect ES** | **incoherence** | | **P value for incoherence** |
| --- | --- | --- | --- | --- | --- |
|  | **RR (95% CI)** | **RR (95% CI)** | **Log IF** | **SE log of IF** |  |
| ATC vs PS | 1.13 (1.04, 1.22) | 0.98 (0.91, 1.05) | 0.139 | 0.055 | 0.011 |
| ATC vs CPAP | 1.15 (1.06, 1.24) | 1.11 (1.04, 1.19) | 0.029 | 0.054 | 0.591 |
| ATC vs T-piece | 1.05 (0.96, 1.15) | 1.17 (1.10, 1.24) | -0.103 | 0.058 | 0.077 |
| CPAP vs PS | 0.69 (0.52, 0.93) | 0.93 (0.90, 0.97) | -0.295 | 0.152 | 0.052 |
| CPAP vs IMV* | 1.00 (0.92, 1.09) | 1.00 (0.85, 1.18) | 0.003 | 0.093 | 0.976 |
| CPAP vs T-piece | 1.00 (0.98, 1.03) | 0.97 (0.88, 1.07) | 0.038 | 0.052 | 0.471 |
| HFNC vs PS | 1.03 (0.91, 1.16) | 0.98 (0.91, 1.06) | 0.048 | 0.073 | 0.509 |
| HFNC vs T-piece* | 1.07 (1.00, 1.14) | 1.09 (0.87, 1.37) | -0.024 | 0.124 | 0.846 |
| IMV vs T-piece | 1.00 (0.91, 1.10) | 0.99 (0.87, 1.13) | 0.004 | 0.081 | 0.959 |
| PAV+ vs PS* | 1.14 (0.89, 1.46) | 0.83 (0.57, 1.21) | 0.316 | 0.247 | 0.201 |
| PAV+ vs T-piece* | 1.05 (0.85, 1.29) | 1.44 (0.92, 2.25) | -0.316 | 0.247 | 0.201 |
| PS vs T-piece | 1.09 (1.06, 1.12) | 0.97 (0.88, 1.08) | 0.108 | 0.055 | 0.048 |

Legend

PS = pressure support; ATC = automatic tube compensation; PAV+ = proportional assist ventilation with load adjustable gain factors; HFNC = High flow nasal cannula; SVT = spontaneous ventilation trial; CPAP = continuous positive airway pressure; IMV = intermittent mandatory ventilation.

**Table S5: SUCRA and Ranking Probabilities for Alternative SBTs on Successful Extubation**

| Treatment | SUCRA | Probability of being the best (%) | Mean Rank |
| --- | --- | --- | --- |
| ATC | 82.5 | 27.6 | 2.4 |
| PS/ATC | 78.2 | 40.1 | 2.7 |
| HFNC | 53.1 | 0.8 | 4.8 |
| PAV+ | 52.1 | 10.7 | 4.8 |
| PS | 51.5 | 0.2 | 4.9 |
| SVT | 42.4 | 14.7 | 5.6 |
| IMV | 39.5 | 6.0 | 5.8 |
| CPAP | 37.1 | 0.0 | 6.0 |
| T-piece | 13.6 | 0.0 | 7.9 |

Legend

PS = pressure support; ATC = automatic tube compensation; PAV+ = proportional assist ventilation with load adjustable gain factors; HFNC = High flow nasal cannula; SVT = spontaneous ventilation trial; CPAP = continuous positive airway pressure; IMV = intermittent mandatory ventilation.

**Table S6 Direct and Indirect Estimates of Effect and Tests for Incoherence for Successful Extubation**

| **Comparison** | **Direct ES** | **Indirect ES** | **incoherence** | | **P value for incoherence** |
| --- | --- | --- | --- | --- | --- |
|  | **RR (95% CI)** | **RR (95% CI)** | **Log IF** | **SE log of IF** |  |
| ATC vs PS | 1.11 (1.01, 1.22) | 1.01 (0.91, 1.11) | 0.094 | 0.070 | 0.182 |
| ATC vs CPAP | 1.07 (0.98, 1.16) | 1.10 (0.99, 1.23) | -0.033 | 0.071 | 0.642 |
| ATC vs T-piece | 1.16 (0.96, 1.40) | 1.12 (1.04, 1.21) | 0.035 | 0.104 | 0.737 |
| CPAP vs PS | 0.99 (0.90, 1.08) | 0.98 (0.91, 1.05) | 0.008 | 0.062 | 0.897 |
| CPAP vs T-piece | 1.03 (0.95, 1.10) | 1.07 (0.96, 1.20) | -0.045 | 0.072 | 0.531 |
| HFNC vs PS | 1.01 (0.95, 1.07) | 0.99 (0.91, 1.08) | 0.013 | 0.053 | 0.806 |
| HFNC vs T-piece* | 1.06 (1.00, 1.12) | 1.10 (0.97, 1.24) | -0.038 | 0.073 | 0.605 |
| PAV+ vs PS* | 1.06 (0.89, 1.26) | 0.89 (0.68, 1.16) | 0.177 | 0.173 | 0.306 |
| PAV+ vs T-piece* | 1.03 (0.89, 1.20) | 1.23 (0.90, 1.68) | -0.177 | 0.173 | 0.306 |
| PS vs T-piece* | 1.07 (1.04, 1.10) | 0.99 (0.88, 1.11) | 0.072 | 0.061 | 0.243 |

Legend

PS = pressure support; ATC = automatic tube compensation; PAV+ = proportional assist ventilation with load adjustable gain factors; HFNC = High flow nasal cannula; SVT = spontaneous ventilation trial; CPAP = continuous positive airway pressure; IMV = intermittent mandatory ventilation.

* All the evidence about these contrasts comes from the trials which directly compare them.

- Network heterogeneity (tau =0.0037); the design-by-treatment interaction model test of network incoherence: p value = 0.118

**Table S7 SUCRA ratings and Ranking Probabilities for Alternative SBT techniques on Reintubation**

| Treatment | SUCRA | Probability of being the best (%) | Mean Rank |
| --- | --- | --- | --- |
| HFNC | 89.1 | 44.0 | 1.9 |
| PS/ATC | 59.8 | 16.0 | 4.2 |
| PAV+ | 59.0 | 3.0 | 4.3 |
| T-piece | 50.2 | 0.0 | 5.0 |
| SVT | 44.6 | 26.4 | 5.4 |
| PS | 41.4 | 0.0 | 5.7 |
| IMV | 39.4 | 10.5 | 5.8 |
| ATC | 38.1 | 0.0 | 5.9 |
| CPAP | 28.3 | 0.0 | 6.7 |

Legend

PS = pressure support; ATC = automatic tube compensation; CPAP = continuous positive airway pressure; HFNC = High flow nasal cannulae; IMV = intermittent mandatory ventilation; PAV+ = proportional assist ventilation with load adjustable gain factors; SVT = spontaneous ventilation trial;

**Table S8 Direct and Indirect Estimates of Effect and Tests for Incoherence for Reintubation**

| **Comparison** | **Direct ES** | **Indirect ES** | **incoherence** | | **P value for incoherence** |
| --- | --- | --- | --- | --- | --- |
|  | **RR (95% CI)** | **RR (95% CI)** | **Log IF** | **SE log of IF** |  |
| ATC vs PS | 1.06 (0.71, 1.60) | 1.01 (0.64, 1.58) | 0.054 | 0.309 | 0.861 |
| ATC vs CPAP | 0.85 (0.45, 1.60) | 0.92 (0.45, 1.87) | -0.076 | 0.484 | 0.875 |
| ATC vs T-piece | 1.10 (0.70, 1.73) | 1.07 (0.72, 1.61) | 0.028 | 0.308 | 0.929 |
| CPAP vs PS | 1.00 (0.24, 4.11) | 1.21 (0.73, 2.01) | -0.190 | 0.766 | 0.804 |
| CPAP vs T-piece | 1.08 (0.60, 1.95) | 1.54 (0.73, 3.22) | -0.349 | 0.477 | 0.464 |
| HFNC vs PS | 0.31 (0.15, 0.68) | 0.38 (0.19, 0.74) | -0.185 | 0.443 | 0.676 |
| HFNC vs T-piece* | 0.37 (0.21, 0.66) | 0.33 (0.11, 0.97) | 0.122 | 0.528 | 0.817 |
| PAV+ vs PS* | 0.72 (0.27, 1.91) | 0.87 (0.19, 4.01) | -0.188 | 0.880 | 0.831 |
| PAV+ vs T-piece* | 0.82 (0.32, 2.11) | 0.68 (0.14, 3.38) | 0.188 | 0.880 | 0.831 |
| PS vs T-piece* | 1.04 (0.90, 1.20) | 1.16 (0.65, 2.07) | -0.111 | 0.305 | 0.716 |

Legend

PS = pressure support; ATC = automatic tube compensation; PAV+ = proportional assist ventilation with load adjustable gain factors; HFNC = High flow nasal cannula; SVT = spontaneous ventilation trial; CPAP = continuous positive airway pressure; IMV = intermittent mandatory ventilation.

**Table S9** **Network Estimates for Alternative SBT techniques on ICU Mortality**

| **PS** | 0.52  (0.25,1.08) | 0.96  (0.35,2.61) | 1.06  (0.22,5.08) | 0.14  (0.01,2.35) | 1.06  (0.84,1.32) |
| --- | --- | --- | --- | --- | --- |
| 1.91  (0.93,3.94) | **ATC** | 1.82  (0.62,5.37) | 2.02  (0.36,11.33) | 0.26  (0.01,4.90) | 2.02  (0.95,4.27) |
| 1.05  (0.38,2.86) | 0.55  (0.19,1.61) | **CPAP** | 1.11  (0.17,7.00) | 0.14  (0.01,2.89) | 1.11  (0.41,2.99) |
| 0.95  (0.20,4.56) | 0.50  (0.09,2.79) | 0.90  (0.14,5.73) | **HFNC** | 0.13  (0.01,3.29) | 1.00  (0.21,4.74) |
| 7.32  (0.43,125.58) | 3.83  (0.20,71.90) | 6.99  (0.35,141.16) | 7.73  (0.30,196.36) | **PAV+** | 7.73  (0.45,131.85) |
| 0.95  (0.76,1.19) | 0.50  (0.23,1.05) | 0.90  (0.33,2.45) | 1.00  (0.21,4.74) | 0.13  (0.01,2.21) | **T-piece** |

Legend

PS = pressure support; ATC = automatic tube compensation; CPAP = continuous positive airway pressure; HFNC = High flow nasal cannula, PAV+ = proportional assist ventilation with load adjustable gain factors.

**Table S10 Network Estimates for Alternative SBT techniques on Hospital Mortality**

| **PS** | 0.75 (0.38,1.48) | 7.22 (0.87,59.99) | 1.00 (0.53,1.88) | 1.21 (0.46,3.18) | 1.09 (0.81,1.49) |
| --- | --- | --- | --- | --- | --- |
| 1.34 (0.67,2.65) | **ATC** | 9.66 (1.04,89.48) | 1.34 (0.53,3.40) | 1.62 (0.49,5.30) | 1.47 (0.69,3.11) |
| 0.14 (0.02,1.15) | 0.10 (0.01,0.96) | **CPAP** | 0.14 (0.02,1.21) | 0.17 (0.02,1.69) | 0.15 (0.02,1.23) |
| 1.00 (0.53,1.88) | 0.75 (0.29,1.90) | 7.21 (0.82,63.14) | **HFNC** | 1.21 (0.40,3.70) | 1.09 (0.62,1.92) |
| 0.83 (0.31,2.18) | 0.62 (0.19,2.02) | 5.96 (0.59,60.01) | 0.83 (0.27,2.53) | **PAV+** | 0.90 (0.34,2.38) |
| 0.91 (0.67,1.24) | 0.68 (0.32,1.45) | 6.59 (0.81,53.59) | 0.91 (0.52,1.60) | 1.11 (0.42,2.91) | **T-piece** |

Legend

PS = pressure support; ATC = automatic tube compensation; CPAP = continuous positive airway pressure; HFNC = High flow nasal cannula, PAV+ = proportional assist ventilation with load adjustable gain factors.

**Table S11** **Network Estimates for Alternative SBT techniques on Most Protracted Mortality**

| **PS** | 0.85 (0.48,1.50) | 1.21 (0.55,2.66) | 1.01 (0.61,1.67) | 1.07 (0.39,2.93) | 1.11 (0.95,1.29) |
| --- | --- | --- | --- | --- | --- |
| 1.18 (0.67,2.10) | **ATC** | 1.44 (0.66,3.12) | 1.19 (0.56,2.55) | 1.27 (0.40,4.02) | 1.31 (0.73,2.35) |
| 0.82 (0.38,1.80) | 0.70 (0.32,1.51) | **CPAP** | 0.83 (0.33,2.08) | 0.88 (0.25,3.14) | 0.91 (0.42,1.99) |
| 0.99 (0.60,1.64) | 0.84 (0.39,1.79) | 1.20 (0.48,3.01) | **HFNC** | 1.06 (0.35,3.24) | 1.10 (0.68,1.78) |
| 0.93 (0.34,2.54) | 0.79 (0.25,2.50) | 1.13 (0.32,4.03) | 0.94 (0.31,2.87) | **PAV+** | 1.03 (0.38,2.82) |
| 0.90 (0.77,1.05) | 0.76 (0.42,1.37) | 1.10 (0.50,2.39) | 0.91 (0.56,1.48) | 0.97 (0.35,2.64) | **T-piece** |

Legend

PS = pressure support; ATC = automatic tube compensation; CPAP = continuous positive airway pressure; HFNC = High flow nasal cannula, PAV+ = proportional assist ventilation with load adjustable gain factors.

**Table S12 Test of Interaction for Risk of Bias Subgroup on Initial SBT Success**

|  |  | RR | 95% CI | | P value |
| --- | --- | --- | --- | --- | --- |
| ATC | Risk of Bias | 0.87 | 0.78 | 0.98 | 0.018 |
|  | _cons | 1.10 | 1.03 | 1.18 | 0.005 |
| PSV/ATC | Risk of Bias | . | . | . | . |
|  | _cons | 1.04 | 0.94 | 1.15 | 0.401 |
| HFNC | Risk of Bias | 1.07 | 0.94 | 1.23 | 0.315 |
|  | _cons | 0.97 | 0.89 | 1.05 | 0.395 |
| IMV | Risk of Bias | . | . | . | . |
|  | _cons | 0.92 | 0.84 | 1.00 | 0.057 |
| PAV+ | Risk of Bias | . | . | . | . |
|  | _cons | 1.02 | 0.85 | 1.24 | 0.813 |
| CPAP | Risk of Bias | 0.98 | 0.89 | 1.08 | 0.711 |
|  | _cons | 0.93 | 0.86 | 1.01 | 0.095 |
| SVT | Risk of Bias | . | . | . | . |
|  | _cons | 0.92 | 0.78 | 1.08 | 0.310 |
| T-piece | Risk of Bias | 1.00 | 0.94 | 1.06 | 0.921 |
|  | _cons | 0.92 | 0.89 | 0.96 | 0.001 |

Legend

PS = pressure support; ATC = automatic tube compensation; HFNC = High flow nasal cannula; IMV = intermittent mandatory ventilation; PAV+ = proportional assist ventilation with load adjustable gain factors; CPAP = continuous positive airway pressure; SVT = spontaneous ventilation trial.

P values in green highlighted cells are for test of interaction; RR's listed in front of "_cons" are RR's for the comparison of Alternative SBT technique versus PSV for studies at low risk of bias.

**Table S13 Test of Interaction for Risk of Bias Subgroup on Successful Extubation**

|  |  | RR | 95% CI | | P value |
| --- | --- | --- | --- | --- | --- |
| ATC | Risk of Bias | 0.84 | 0.73 | 0.98 | 0.026 |
|  | _cons | 1.19 | 1.06 | 1.34 | 0.004 |
| PSV/ATC | Risk of Bias | . | . | . | . |
|  | _cons | 1.07 | 0.93 | 1.23 | 0.340 |
| CPAP | Risk of Bias | 0.97 | 0.87 | 1.09 | 0.636 |
|  | _cons | 0.99 | 0.91 | 1.07 | 0.803 |
| HFNC | Risk of Bias | 1.01 | 0.91 | 1.13 | 0.825 |
|  | _cons | 0.99 | 0.90 | 1.09 | 0.882 |
| IMV | Risk of Bias | . | . | . | . |
|  | _cons | 0.95 | 0.76 | 1.19 | 0.661 |
| PAV+ | Risk of Bias | . | . | . | . |
|  | _cons | 0.99 | 0.87 | 1.14 | 0.908 |
| SVT | Risk of Bias | . | . | . | . |
|  | _cons | 0.96 | 0.74 | 1.24 | 0.735 |
| T-piece | Risk of Bias | 1.03 | 0.98 | 1.09 | 0.252 |
|  | _cons | 0.93 | 0.89 | 0.96 | 0.000 |

Legend

PS = pressure support; ATC = automatic tube compensation; CPAP = continuous positive airway pressure; HFNC = High flow nasal cannula; IMV = intermittent mandatory ventilation; PAV+ = proportional assist ventilation with load adjustable gain factors; SVT = spontaneous ventilation trial.

P values in green highlighted cells are for test of interaction; RR's listed in front of "_cons" are RR's for the comparison of Tx versus PSV for studies at low risk of bias.

**Table S14 Test of Interaction for Risk of Bias Subgroup on Reintubation**

|  |  | RR | 95% CI | | P value |
| --- | --- | --- | --- | --- | --- |
| ATC | Risk of Bias | 0.97 | 0.50 | 1.86 | 0.915 |
|  | _cons | 1.02 | 0.59 | 1.75 | 0.941 |
| PSV/ATC | Risk of Bias | . | . | . | . |
|  | _cons | 0.67 | 0.12 | 3.82 | 0.649 |
| CPAP | Risk of Bias | 1.81 | 0.66 | 4.94 | 0.250 |
|  | _cons | 0.94 | 0.49 | 1.81 | 0.858 |
| HFNC | Risk of Bias | 0.66 | 0.21 | 2.08 | 0.478 |
|  | _cons | 0.44 | 0.20 | 0.98 | 0.044 |
| IMV | Risk of Bias | . | . | . | . |
|  | _cons | 2.08 | 0.04 | 102.23 | 0.713 |
| PAV+ | Risk of Bias | . | . | . | . |
|  | _cons | 0.78 | 0.33 | 1.86 | 0.575 |
| SVT | Risk of Bias | . | . | . | . |
|  | _cons | 1.82 | 0.01 | 436.05 | 0.831 |
| T-piece | Risk of Bias | 0.90 | 0.67 | 1.19 | 0.460 |
|  | _cons | 1.01 | 0.83 | 1.23 | 0.903 |

Legend

PS = pressure support; ATC = automatic tube compensation; CPAP = continuous positive airway pressure; HFNC = High flow nasal cannula; IMV = intermittent mandatory ventilation; PAV+ = proportional assist ventilation with load adjustable gain factors; SVT = spontaneous ventilation trial.

P values in green highlighted cells are for test of interaction; RR's listed in front of "_cons" are RR's for the comparison of Tx versus PSV for studies at low risk of bias.

**Figure S1 Network Plot of Alternative SBT Techniques on ICU Mortality**


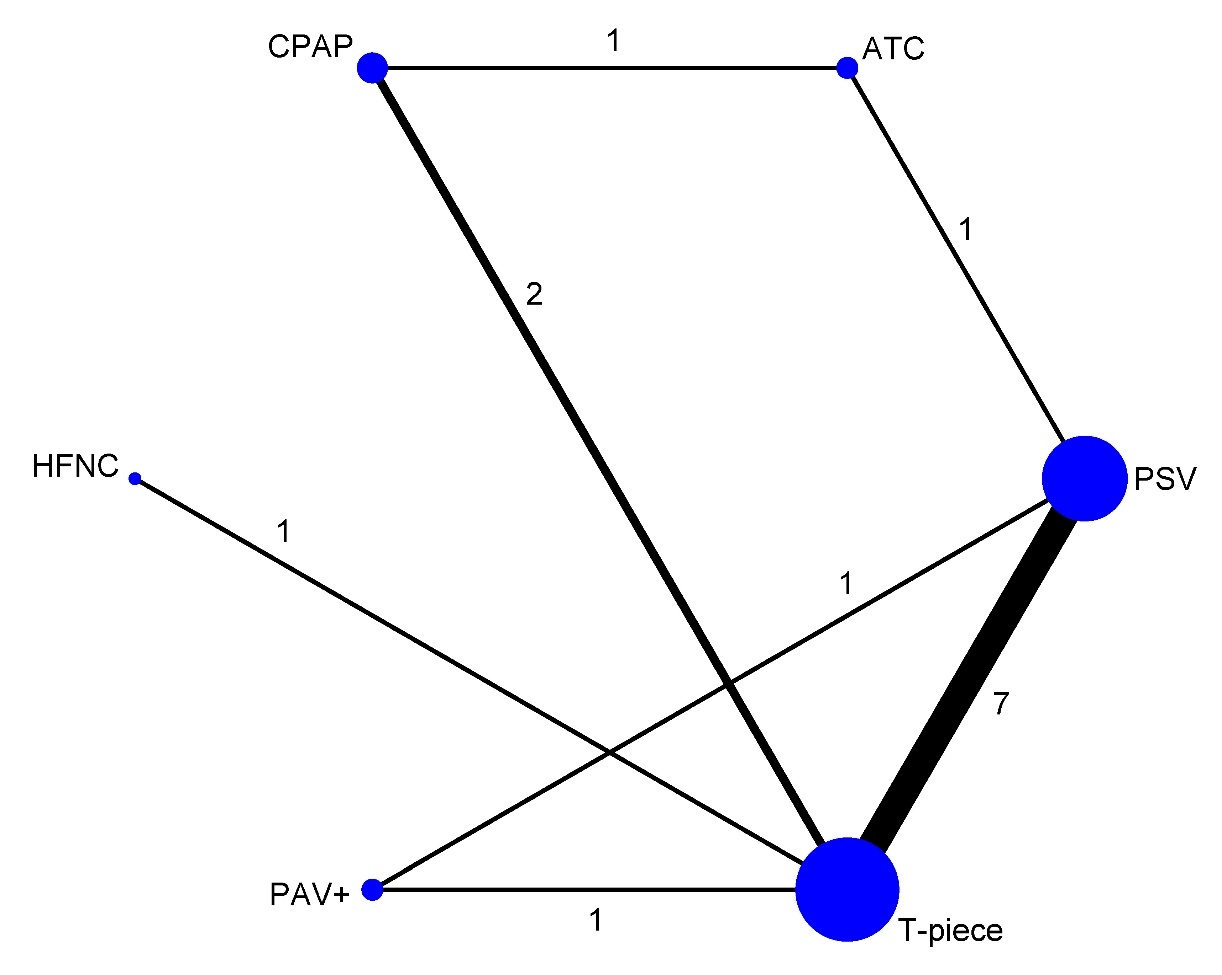


**Figure S2 Network Plot of Alternative SBT Techniques on Hospital Mortality**


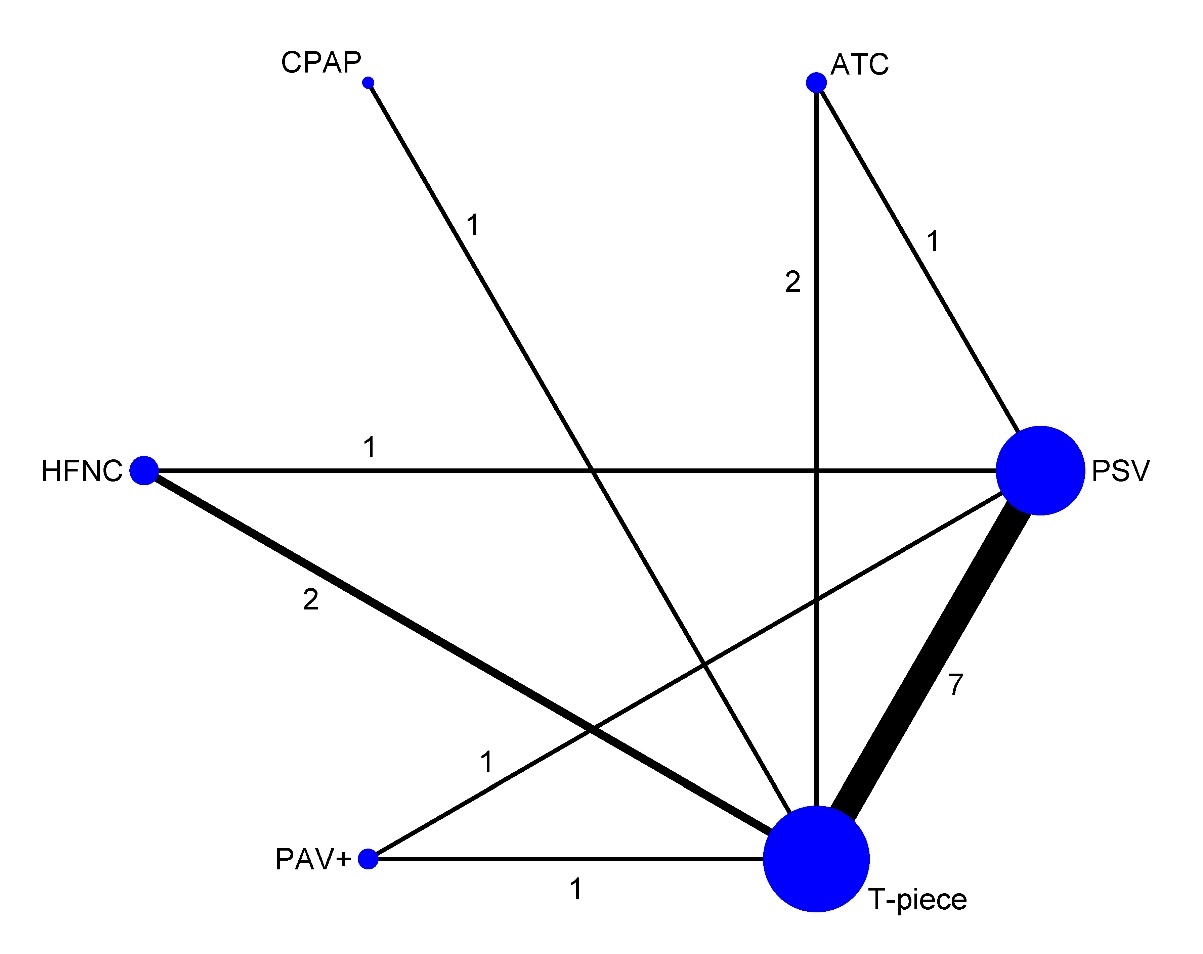


**Figure S3 Network Plot of Alternative SBT Techniques on Most Protracted Mortality**


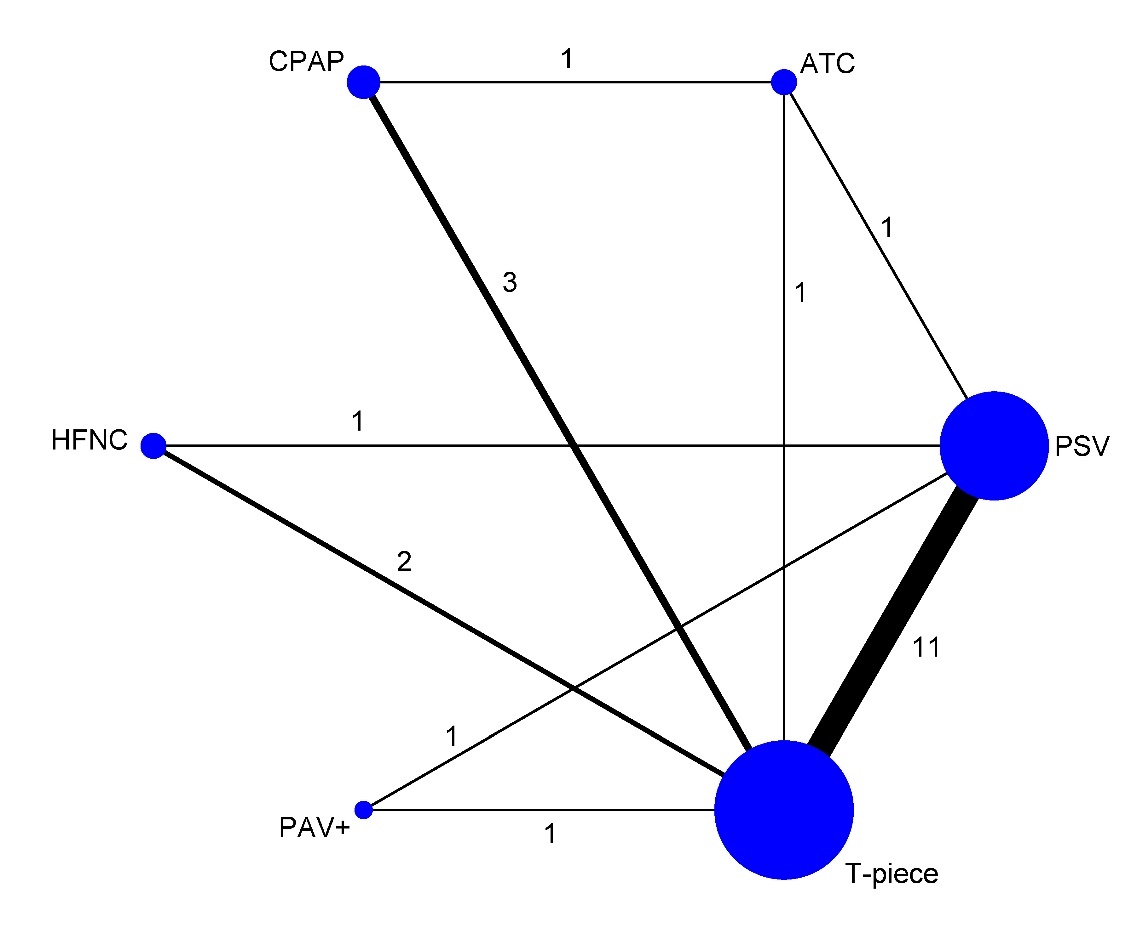

Supplement: Supplementary file 1 — Additional file 1 (DOCX 343 kb) [file 13054_2024_4958_MOESM1_ESM.docx]
